# Lifetime fitness consequences of early‐life ecological hardship in a wild mammal population

**DOI:** 10.1002/ece3.2747

**Published:** 2017-02-12

**Authors:** Harry H. Marshall, Emma I. K. Vitikainen, Francis Mwanguhya, Robert Businge, Solomon Kyabulima, Michelle C. Hares, Emma Inzani, Gladys Kalema‐Zikusoka, Kenneth Mwesige, Hazel J. Nichols, Jennifer L. Sanderson, Faye J. Thompson, Michael A. Cant

**Affiliations:** ^1^ Centre for Ecology and ConservationUniversity of ExeterCornwallUK; ^2^ Banded Mongoose Research ProjectQueen Elizabeth National ParkKasese DistrictUganda; ^3^ Conservation Through Public HealthEntebbeUganda; ^4^ School of Natural Science and PsychologyLiverpool John Moores UniversityLiverpoolUK

**Keywords:** early‐life, ecological variability, fitness effects, life‐history strategy, mammal, sex‐specific

## Abstract

Early‐life ecological conditions have major effects on survival and reproduction. Numerous studies in wild systems show fitness benefits of good quality early‐life ecological conditions (“silver‐spoon” effects). Recently, however, some studies have reported that poor‐quality early‐life ecological conditions are associated with later‐life fitness advantages and that the effect of early‐life conditions can be sex‐specific. Furthermore, few studies have investigated the effect of the variability of early‐life ecological conditions on later‐life fitness. Here, we test how the mean and variability of early‐life ecological conditions affect the longevity and reproduction of males and females using 14 years of data on wild banded mongooses (*Mungos mungo*). Males that experienced highly variable ecological conditions during development lived longer and had greater lifetime fitness, while those that experienced poor early‐life conditions lived longer but at a cost of reduced fertility. In females, there were no such effects. Our study suggests that exposure to more variable environments in early life can result in lifetime fitness benefits, whereas differences in the mean early‐life conditions experienced mediate a life‐history trade‐off between survival and reproduction. It also demonstrates how early‐life ecological conditions can produce different selection pressures on males and females.

## 1 Introduction

From life's school of war: what does not kill me makes me stronger. Friedrich Nietzsche (1889), *Twilight of the Idols*

The principle that a good start in life can have many advantages later on is well recognized in ecology and evolution (Lindström, 1999; Monaghan, 2008). Indeed, numerous ecological studies have shown that favorable early‐life ecological conditions have positive “silver‐spoon” effects on individuals’ later‐life survival (Cartwright, Nicoll, Jones, Tatayah, & Norris, 2014; Reid, Bignal, Bignal, McCracken, & Monaghan, 2003; Van de Pol, Bruinzeel, Heg, Van der Jeugd, & Verhulst, 2006; Wong & Kölliker, 2014) and reproduction (Balbontin & Moller, 2015; Douhard et al., 2014; Hayward, Rickard, & Lummaa, 2013; Millon, Petty, Little, & Lambin, 2011; Nussey, Kruuk, Morris, & Clutton‐Brock, 2007).

Recently, however, there has been growing evidence that the effect of early‐life ecological conditions on later‐life fitness is not so straightforward. First, some studies have found indications that poor — rather than good — early‐life ecological conditions can have favorable effects on fertility or survival in later life (Garratt et al., 2015; Rubenstein et al., 2016; Wilkin & Sheldon, 2009). For example, male superb starlings (*Lamprotonis superbus*) which hatched in a low prebreeding rainfall year (an indicator of poor ecological conditions in this system) had lower rates of DNA methylation which in turn was associated with a greater probability of breeding in later life (Rubenstein et al., 2016). There is also evidence that, despite an overall positive effect of favorable early‐life ecological conditions on fitness, cohorts of male great tits (*Parus major*) born in poor years live longer and have greater reproductive success (Wilkin & Sheldon, 2009). It has been proposed, but not tested, that this relationship could arise because of increased maternal investment during harsh periods (Rubenstein et al., 2016), or stronger selection during periods of hardship (Garratt et al., 2015; Wilkin & Sheldon, 2009). These findings may also relate to a widely recognized phenomenon in laboratory animals and humans that dietary restriction, without malnutrition, has beneficial effects such as increased longevity (e.g., Colman et al., 2009; Fontana, Meyer, Klein, & Holloszy, 2004; Masoro, 2006; McCay, Crowell, & Maynard, 1935; Zhang et al., 2013). Second, there is evidence that individuals can adjust life‐history trajectories to achieve similar lifetime fitness, despite differing early‐life conditions (Gluckman, Hanson, & Spencer, 2005; Monaghan, 2008; Nettle & Bateson, 2015; Taborsky, 2006). For example, Seychelles warblers (*Acrocephalus sechellensis*) born into unfavorable ecological conditions have shorter lifespans, but also start to breed earlier with no difference in the length of the reproductive lifespan compared to individuals born during periods of abundance (Cartwright et al., 2014; Hammers, Richardson, Burke, & Komdeur, 2013). Third, early‐life effects in later life are often manifested in one sex but not the other (Ancona & Drummond, 2013; Garratt et al., 2015; Kruuk, Clutton‐Brock, Rose, & Guinness, 1999; Millon et al., 2011; Rubenstein et al., 2016; Wilkin & Sheldon, 2009; Wong & Kölliker, 2014). In red deer (*Cervus elaphus*), females that experience lower temperatures in early life have reduced reproductive success, but males show no such relationship (Kruuk et al., 1999). In the European earwig (*Forficula auricularia*), low food availability during development reduces lifetime reproductive success in females but not males (Wong & Kölliker, 2014).

Ecological conditions in a given space and time can be characterized by their average value and also by how much they vary around this average. Despite this, previous studies have (implicitly or explicitly) tended to focus on the effect of the average early‐life ecological conditions on later‐life, and not considered the effect of their variability. Yet, environmental variability can have profound implications on ecological and evolutionary processes (e.g., Botero, Weissing, Wright, & Rubenstein, 2014). For example, theoretical work has shown that direction and strength of the correlation between maternal and offspring phenotype, that is, the maternal effect, should be affected by the magnitude and predictability of the environment's fluctuations (Hoyle & Ezard, 2012; Kuijper & Hoyle, 2015). There are also instructive examples from studies of human medicine and psychology suggesting that the variability of conditions experienced during early‐life may have important later‐life implications. First, exposure to a narrower variety of pathogens during childhood can impair the development of the immune system, resulting in inappropriate immune responses in adulthood and the dramatic increase in allergic diseases observed in Western societies over the last few decades (the “hygiene hypothesis”: Strachan, 1989, 2000; Wills‐Karp, Santeliz, & Karp, 2001; Yazdanbakhsh, Kremsner, & van Ree, 2002). Second, exposure to a wide variety of psychological stressors during childhood may have important effects on aspects of cognitive development, with evidence that a stressful childhood, while impinging on health and wellbeing, may also produce adults who perform better in cognitive tasks which involve an element of stress (Frankenhuis & de Weerth, 2013; Frankenhuis, Panchanathan, & Nettle, 2015).

**Figure 1:**
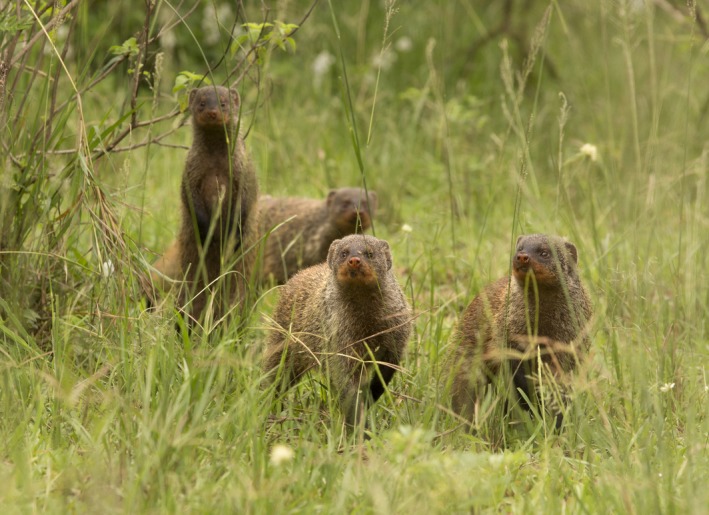
Banded mongooses (*Mungos mungo*) moving as a group and inspecting what lies ahead. Photo credit: Feargus Cooney

In this study, we test the sex‐specific effects of the mean and variability of early‐life ecological conditions on later‐life survival and reproductive success in wild banded mongooses (*Mungos mungo*, Figure 1). We use a 14‐year data set describing ecological conditions (measured by annual rainfall) and mongoose body condition, survival and reproduction to address two questions. First, how do early‐life ecological conditions influence early‐life social environment, body condition and survival? This question is stimulated by previous studies which have argued that the effects of early‐life ecological conditions on later life may result from changes in the amount of care received during development (Rubenstein et al., 2016) or the quality of offspring that survive to adulthood (Garratt et al., 2015; Wilkin & Sheldon, 2009). Second, how do early‐life ecological conditions affect lifetime survival and reproductive success? We test whether the effects of both the mean and variability of early‐life ecological conditions on later‐life survival and reproduction represent a classic silver‐spoon effect (Nussey et al., 2007; Reid et al., 2003; Van de Pol et al., 2006) or support more recent indications that poor early‐life conditions can be advantageous (Garratt et al., 2015; Rubenstein et al., 2016; Wilkin & Sheldon, 2009). We also test whether the later‐life effects we find (1) are due to a life‐history trade‐off between survival and reproduction (Cartwright et al., 2014; Hammers et al., 2013) and (2) differ between males and females (Kruuk et al., 1999; Millon et al., 2011; Wilkin & Sheldon, 2009).

## 2 Materials and Methods

### 2.1 Study system

We conducted our study between September 1999 and March 2014 on a long‐term study population of banded mongooses on the Mweya Peninsula in Queen Elizabeth National Park, Uganda (0°12′S, 27°54′E). Cant, Vitikainen, and Nichols (2013) and Cant, Nichols, Thompson, and Vitikainen (2016), and references therein, provide detailed descriptions of our study site and banded mongoose biology. Here, we provide information about both specific to this study.

Banded mongooses (Figure 1) are diurnal carnivores (<2 kg) that live in stable, mixed‐sex groups of around 10–30 individuals and whose diet mainly consists of small invertebrates (Rood, 1975). Individuals sexually mature around the age of 1 year (Cant et al., 2016) and all individuals within a group reproduce to some extent, though contributions to reproduction are skewed toward older individuals (Nichols, Amos, Cant, Bell, & Hodge, 2010; Nichols, Bell, Hodge, & Cant, 2012). Their average lifespan is around 3.5 years (males = 42 months, females = 38 months, max = 149 months, Cant et al., 2016), and survival rates are constant across all ages (Cant et al., 2016; Marshall et al., 2016). At our equatorial study site reproduction occurs all year round and is not synchronized between groups. Reproduction is, however, highly synchronized within groups: around four times per year, all pregnant females in a group give birth in an underground den to a large communal litter, usually on the same morning (Cant, 2000; Hodge, Bell, & Cant, 2011). Pups remain in the den for approximately their first 30 days, after which they move with the rest of the group and are cared for by adult “escorts” for around a further 60 days (Gilchrist, 2004; Gilchrist & Russell, 2007). We are able to individually recognize the mongooses in our study population using unique hair‐shave patterns or colored collars. Radio collars weighing 26–30 g (Sirtrack Ltd, Havelock North, New Zealand) with a 20‐cm whip antenna (Biotrack Ltd, Dorset, UK) are fitted to one to two individuals in each group to allow them to be located. Most individual are trained to step onto an electronic balance in return for a small milk reward. Two groups have access to human refuse (Otali & Gilchrist, 2004) and so were excluded from this study (and also a previous complementary study investigating the effects of ecological conditions during adulthood, Marshall et al., 2016).

### 2.2 Data collection

#### 2.2.1 Ecological conditions

**Figure 2:**
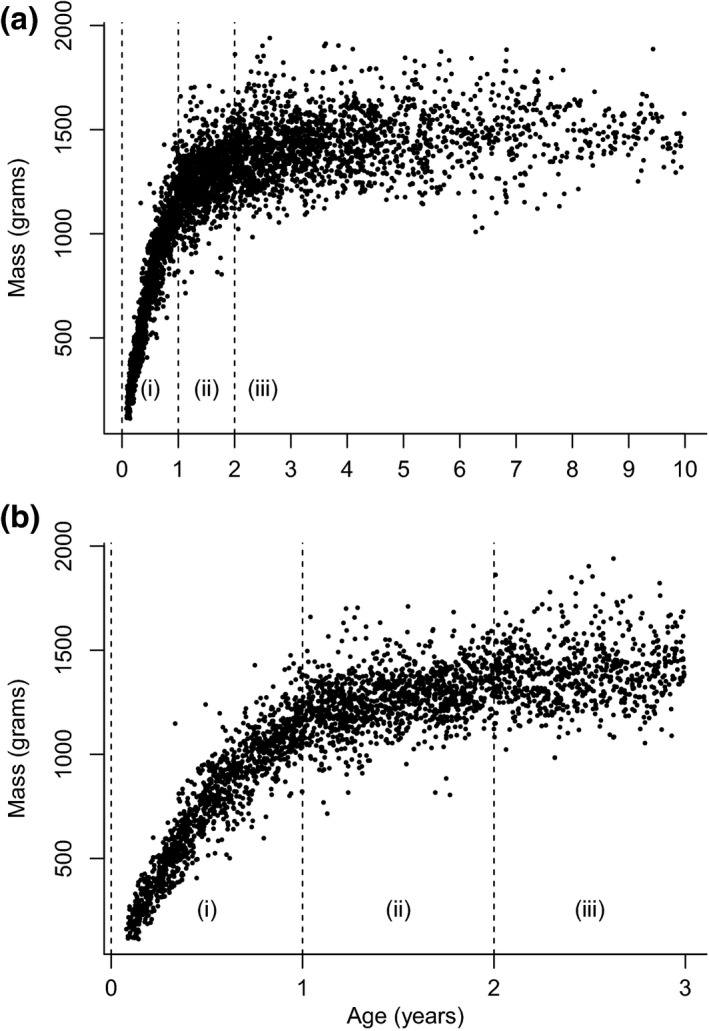
Banded mongoose mass change with age. Panel (a) shows all data and panel (b) zooms in on data from individuals between the ages of 0 and 3 years. In both panels, the vertical dotted lines divide the data into masses from individuals aged 0 to 1 year (zone i), 1 to 2 years (zone ii) and over 2 years (zone iii)

**Figure 3:**
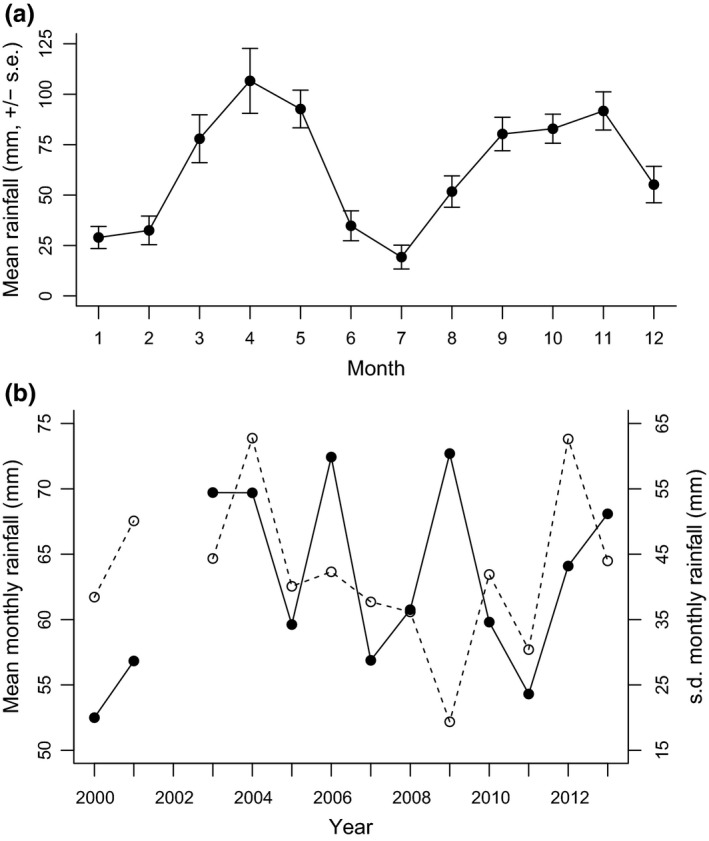
The rainfall pattern (a) within and (b) between years at our study site: the Mweya Peninsula, Uganda. Panel (a) shows the mean (± standard error) rainfall recorded in each month (*n* = 152 months, month 1 = January). Panel (b) shows the mean (filled circles and solid line) and standard deviation (open circles and dashed line) of the monthly rainfall within each year. Rainfall data for 2002 are incomplete and so not shown

We collected climate data daily from a weather station situated centrally at our study site. We selected rainfall as our measure of ecological conditions since it is relatively variable at Mweya (mean monthly rainfall ± *SD* = 61 ± 41 mm, *n* = 152 months), while temperature is reasonably constant (mean of monthly mean maximum daily temperature ± *SD* = 29.5 ± 1.5°C, *n* = 162 months) (Marshall et al., 2016). We defined an individual's “early life” to be its first year of development based on growth and age at sexual maturity. Banded mongooses’ major growth phase occurs in their first year (zone i in Figure 2), and 1 year is also the approximate age at which females become regular breeders and males first obtain paternity (Cant et al., 2016). In addition, the pattern of rainfall at our study site operates over a 12‐month period: two wet seasons, one shorter (March–May) and one longer (August–December), divided by two dry seasons (January–February and June–July; Figure 3a, Marshall et al., 2016). Consequently, we used the mean and standard deviation of the monthly rainfall in mongooses’ first year of life as our measure of the mean and variability of early‐life ecological conditions (Figure 3b). At our study site, the mean and standard deviation of monthly rainfall within a year are not correlated (Pearson's *r* = .11, *p* = .73, *n* = 13 years). This standard deviation is, however, positively correlated with the maximum monthly rainfall in the year (*r* = .91, *p* < .001, *n* = 13) and negatively correlated with the minimum rainfall in the year (*r* = −.71, *p* = .007, *n* = 13). Thus, a high standard deviation in monthly rainfall over a 12‐month period indicates a period with greater extremes of low and high monthly rainfall. As you would expect, mean monthly rainfall in a year is moderately positively correlated with both the minimum (*r* = .46, *p* = .12, *n* = 13) and maximum (*r* = .32, *p* = .28, *n* = 13) monthly rainfall.

We further explored the ecological relevance of rainfall at our study site by measuring its effect on invertebrates (banded mongooses’ predominant food source; Rood, 1975) using monthly pitfall trap transects set between August 2013 and February 2015. At the end of each month, we set pitfall traps on 40‐m transects at four randomly chosen locations. Each transect consisted of five pitfall traps set at 10‐m intervals along the transect line. Pitfall traps consisted of smooth‐side plastic drinking cups (9 cm tall with circular tops 7 cm in diameter) buried so that their rims were flush with the level of the ground. These were filled with water to c. 3 cm deep and a drop of detergent to break the water's surface tension and ensure captured invertebrates fell into the water rather than being caught on its surface. The traps were collected after 24 hr, and their contents were sieved out and frozen in ziplock bags in a −20°C freezer. At a later date, the contents of the pitfall traps in each transect were then sorted and the invertebrates were identified to order level. Following Rood (1975), we identified mongoose prey as those invertebrates belonging to the orders Blattodea, Coleoptera, Diplopoda, Formicidae, and Orthoptera. We measured the abundance and diversity of invertebrate prey collected in each transect. Abundance was measured as the total number of individuals in all five orders. Diversity was calculated using the Shannon–Weiner index of diversity to measure the distribution of these individuals across the five orders. The Shannon–Weiner index (*H*) for a particular transect was calculated as:

$$H=-\sum_{i=1}^{s}\;p_{i}\;\text{ln}\;p_{i}.$$

Here, *s* is the total number of orders and *p*_*i*_ is the proportional abundance of the *i*th order in that transect. Our sampling yielded measures of mongoose invertebrate prey abundance and diversity from 36 transects from 9 months (some monthly samples were lost).

#### 2.2.2 Banded mongooses

We visited our study groups for at least 20 min every 1–3 days, during which we noted the presence or absence of individuals. We could distinguish between absences due to dispersal from the group and due to death since in banded mongooses dispersal involves the simultaneous eviction of multiple individuals from the group, often with a conspicuous period of aggression within the group beforehand (Cant, Hodge, Bell, Gilchrist, & Nichols, 2010; Thompson et al., 2016). In contrast, death involves the permanent disappearance of single individuals. We were able to weigh most individuals once a week in the morning before foraging started by training them to step on electronic scales in return for a small milk reward. We identified female pregnancy by visual swelling of the abdomen and confirmed this by palpation and ultrasound scans during trapping (Cant, 2000; Inzani et al., 2016). Births occur overnight in an underground den and were identified by the absence of pregnant females the following morning and a subsequent change in their body shape and mass loss (Cant, 2000). To assign parentage, DNA was extracted from 2‐mm skin samples taken from individuals when they were first trapped (either as newly emerged pups or newly arrived immigrants). This DNA was then genotyped using a panel of 43 polymorphic microsatellite markers (see further details of DNA analysis and parentage assignment in Sanderson, Wang, Vitikainen, Cant, & Nichols, 2015). See Hodge (2007) and Jordan, Mwanguhya, Kyabulima, Ruedi, and Cant (2010) for further details of the trapping procedure.

To answer part of our first question about whether early‐life ecological conditions affect early‐life social environment, we measured the amount of social care an individual received as a pup and the social rank of their parents. The amount of escorting (social care) a mongoose pup receives has been shown to affect its condition and survival during development and later‐life reproduction (Gilchrist, Otali, & Mwanguhya, 2004; Hodge, 2005). Similarly, parental social rank has been shown to influence individuals’ access to resources and growth in early‐life, and reproduction in later‐life in other systems (Altmann & Alberts, 2005; Charpentier, Tung, Altmann, & Alberts, 2008; Huchard et al., 2013). We measured the amount of escorting received by a pup as the proportion of group visits they were observed being escorted by an adult during the escorting period. Pups were defined as being escorted if they were within 30 cm of the same adult for more than 50% of the group visit (Gilchrist, 2004; Sanderson et al., 2014). Social dominance increases with age in banded mongooses (e.g., Cant, Nichols, Johnstone, & Hodge, 2014; Nichols et al., 2010) and so parental rank was measured as the parent's age‐rank within the group on the day of an individual's birth.

### 2.3 Statistical analyses

#### 2.3.1 Effects of ecological conditions on invertebrate prey abundance and diversity

Prior to our main analyses, we explored the ecological relevance of our rainfall measures. To do this, we fitted models predicting invertebrate prey abundance and species diversity in each monthly pitfall trap transect. In these models, we included the following fixed effects: the total rainfall in the past 30 days to test for shorter‐term rainfall effects, the mean and standard deviation of the monthly rainfall in the past 12 months to test for longer‐term rainfall effects and the quadratic terms of this mean and standard deviation to test for any nonlinear effects. We included collection month as a random intercept. The abundance data were overdispersed but not zero‐inflated so we also included an observation‐level random effect in the abundance model and fitted it using a Poisson log‐normal error structure and log link function (Harrison, 2014). The diversity model was fitted using a normal error structure and its residuals checked to ensure they were normally distributed with a homogeneous variance.

#### 2.3.2 Effects of early‐life ecological conditions on banded mongooses

Mirroring our research questions, we conducted our analyses of the effect of early‐life ecological conditions in two stages: (1) effects during early‐life and (2) lifetime fitness effects, fitting our models to males and females separately. Banded mongooses are fully grown and reach sexual maturity between the ages of 1 and 2 years (zone ii in Figure 2; Cant et al., 2016). Therefore, in stage 2 of our analyses, we only included individuals who had survived to 2 years old to ensure we only included fully developed individuals (zone iii in Figure 2). Table 1 lists the models we fitted in each stage and details how the response variable in each model was measured, the sample sizes used, the random effects included and the models’ error structure and link functions. In all models, we fitted the mean and standard deviation of the monthly rainfall in an individual's first year of life as fixed effects. To test for the possibility of a life‐history trade‐off between survival and reproduction, our models of lifespan also included whether an individual had successfully reproduced in their lifetime (0/1) and the interaction between this and the first‐year rainfall variables. Our model of adult mass also included an individual's age as a control variable, while our model predicting the amount of social care received as a pup also included the ratio of adults (potential escorts) to pups in the group as a control variable. Banded mongoose reproduction is synchronized within groups, but not between groups, and in our equatorial study population reproduction occurs all year round. Consequently, population‐wide cohort effects, which can lead to spurious findings in the analysis of life‐history traits (Grosbois et al., 2008; Murray, 2006), are not expected in this system. Within‐group reproductive synchrony does form cohorts of individuals born in the same communal litter who all experience the same early‐life ecological conditions, and we controlled for this in stage 1 of our analysis by including breeding attempt as a random effect in our models (Table 1). It was not necessary to control for within‐group cohort effects in stage 2 of our analyses because the high mortality rate in our population (e.g., 57% mortality between 1 and 3 months old, Hodge et al., 2009; see full survival curve in Cant et al., 2016) meant that individuals included in these analyses were almost entirely from unique breeding attempts.

**Table 1:** Details of the models fitted in each stage of our analyses. Samples sizes are shown for males (M) and females (F) as models were fitted separately to data from each sex. All models included the mean and standard deviation of the monthly rainfall in an individual's first year of life as fixed effects

Models predicting how early‐life ecological conditions affect:	Measured as	Sample size						Model fitting		
		Individuals		Packs		Breeding attempts		Random effects	Error structure	Link function
		M	F	M	F	M	F			
Stage 1: effects during early life										
Maternal rank	The age‐rank of an individual's mother in the pack at the time of their birth	104	84	10	9	63	48	Breeding attempt, pack	Poisson	Log
Paternal rank	The age‐rank of an individual's father in the pack at the time of their birth	86	63	10	9	53	43	Breeding attempt, pack	Poisson	Log
Social care received^1^	The proportion of group visits an individual was observed being escorted as a pup	49	41	5	5	27	21	Breeding attempt, pack	Binomial	Logit
Mass at 1 year	Individual's mass (g) at 1 year old (±30 days)	47	15	6	2	27	12	Breeding attempt, pack	Normal	Identity
Survival to 1 year	Binary denoting if an individual survived to 1 year old	357	300	14	13	132	117	Breeding attempt, pack	Binomial	Logit
Stage 2: lifetime fitness effects										
Body condition^a^^,^ 2	Mass (g)	80	41	8	8			Individual, pack	Normal	Identity
Successful reproduction in lifetime	Binary denoting if an individual was assigned as parent to a pup during their lifetime	61	43	9	8			Pack	Binomial	Logit
Relative fertility of successful reproducers	Proportion of all genotyped pups born while an individual was resident in a pack that they were assigned as parent to	19	22	5	5			Pack	Binomial	Logit
Lifetime reproductive success	Total number of pups an individual was assigned in its lifetime	58	37	9	8			Pack	Negative binomial	Log
Lifespan^3^	Age (years) at which an individual died	60	43	9	8			Pack, observation‐level	Poisson log‐normal	Log

^a^Models fitted to 174 and 90 records of male and female masses, respectively. Also included as fixed effects: ^1^number of adults (potential escorts) in the group, ^2^an individual's age, ^3^whether an individual had successfully reproduced in their lifetime (0/1) and the interaction between this and the first‐year rainfall variables.

We conducted our analyses using generalized linear mixed effect models (GLMMs). The residuals of models fitted using normal error structures (mass at 1 year old and adult mass) were checked to ensure they were normally distributed with constant variance. Models fitted to count data used a Poisson error structure unless they were overdispersed. Where this occurred, following Harrison (2014), models were fitted using a Poisson log‐normal error structure and an observation‐level random effect (lifespan models) unless the data were zero‐inflated in which case a negative binomial error structure was used (lifetime reproductive success models). We used the same model selection procedure as our complementary study of the effect of ecological conditions during adulthood on banded mongooses (Marshall et al., 2016). We used likelihood ratio tests, comparing the full model to a model without a particular variable, to test the significance of this variable's effect (Forstmeier & Schielzeth, 2011). Where interactions did not have a significant effect, we dropped these from our final model to allow us to test the significance of the main effects in these nonsignificant interactions (Engqvist, 2005). We did not reduce our model further due to the issues with stepwise model reduction techniques (Forstmeier & Schielzeth, 2011; Mundry & Nunn, 2009; Whittingham, Stephens, Bradbury, & Freckleton, 2006). Correlations between variables fitted in models as fixed effects — including the correlation between the mean and standard deviation of the monthly rainfall in a mongoose's first year — were lower than the levels previously shown to cause model fitting issues such as variance inflation in effect estimates (max *r* = .36, Freckleton, 2011). We performed all analyses in R (R Core Team, 2014), fitting GLMMs using the lme4 package (Bates, Maechler, Bolker, & Walker, 2014).

## 3 Results

### 3.1 Effects of ecological conditions on invertebrate prey abundance and diversity

**Figure 4:**
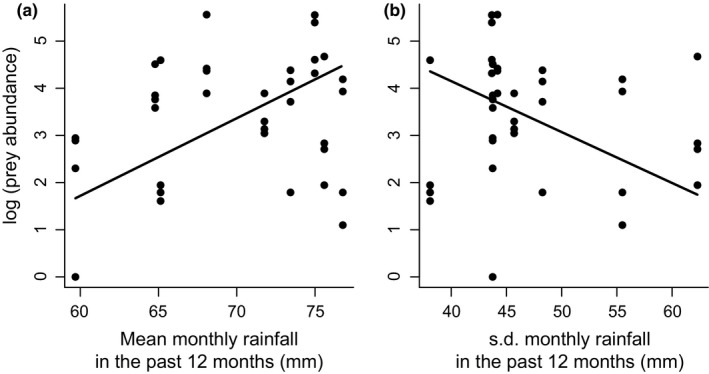
The effect of (a) the mean and (b) the standard deviation of monthly rainfall in the past 12 months on invertebrate prey abundance. Note the log scale on the *y* axis

Invertebrate prey abundance was predicted by the pattern of rainfall in the preceding 12 months: prey abundance increased with the mean monthly rainfall in the previous 12 months (β ± *SE* = 0.16 ± 0.06, χ^2^ = 5.70, *p* = .017; Figure 4a) and decreased with the standard deviation of monthly rainfall in the previous 12 months (β ± *SE* = −0.11 ± 0.04, χ^2^ = 4.50, *p* = .034; Figure 4b). Invertebrate prey abundance was not affected by the amount of rainfall in the past 30 days (χ^2^ = 2.14, *p* = .14; model intercept ± *SE* = −2.53 ± 2.96). There was no evidence for a quadratic relationship between invertebrate abundance and either rainfall measure (mean rainfall: χ^2^ = 0.79, *p* = .38; *SD* rainfall: χ^2^ = 2.96, *p* = .09). Invertebrate prey diversity was not predicted by any rainfall measure (linear terms, mean rainfall: χ^2^ = 3.19, *p* = .07, *SD*, rainfall: χ^2^ = 1.99, *p* = .16; quadratic terms, mean rainfall: χ^2^ = 0.24, *p* = .63, *SD* rainfall: χ^2^ = 1.35, *p* = .24; rainfall in last 30 days: χ^2^ = 0.71, *p* = .40; model intercept ± *SE* = 1.82 ± 0.96). It appears, therefore, that invertebrate prey abundance at our site is influenced by longer‐, rather than shorter‐term patterns in rainfall. This is consistent with other studies showing that ground‐dwelling invertebrates can have long life cycles (e.g., 2 years in millipedes, Lewis, 1971) and that their abundance can be more greatly influenced by longer‐term rainfall patterns (Kwok, Wardle, Greenville, & Dickman, 2016).

### 3.2 Effects of early‐life ecological conditions on banded mongooses

Early‐life ecological conditions, as measured by the mean and variability of rainfall in a mongoose's first year, did not predict the amount of social care received in their first year or their survival to 1 year old (Table 2). Early‐life conditions did not have a significant effect on body mass at 1 year old; however, there was a trend for males born in more variable years to be heavier at 1 year old (*p* = .06, Table 2).

**Table 2:** Models predicting the effect of early‐life ecological conditions on early‐life social environment, body condition and survival. Parameter estimates (±standard errors) for models fitted to males and females are shown, along with likelihood‐ratio chi‐square statistics and *p*‐values

Model predicting	Explanatory variable	Males				Females			
		β	*SE*	χ^2^	*p*	β	*SE*	χ^2^	*p*
Maternal rank	Intercept	0.34	0.67			0.64	0.74		
	Mean monthly rainfall in first year	0.004	0.01	0.17	.68	0.01	0.01	0.31	.58
	*SD* monthly rainfall in first year	0.01	0.01	2.00	.16	−0.004	0.01	0.49	.49
Paternal rank	Intercept	0.40	0.75			0.17	0.87		
	Mean monthly rainfall in first year	0.01	0.01	1.33	.25	0.01	0.01	0.47	.49
	*SD* monthly rainfall in first year	−0.01	0.01	0.66	.42	0.002	0.01	0.10	.75
Social care received	Intercept	−0.22	2.91			3.37	2.04		
	Mean monthly rainfall in first year	0.02	0.04	0.33	.56	−0.04	0.03	2.15	.14
	*SD* monthly rainfall in first year	−0.02	0.04	0.24	.63	−0.02	0.03	0.42	.51
	Ratio of adults to pups in the group	−0.02	0.14	0.02	.90	−0.18	0.11	2.12	.15
Mass at 1 year old	Intercept	1,329.35	248.83			1,082.51	222.98		
	Mean monthly rainfall in first year	−4.48	2.90	2.33	.13	0.40	3.64	0.04	.84
	*SD* monthly rainfall in first year	3.72	1.95	3.68	.06	0.20	1.38	0.02	.88
Survival to 1 year	Intercept	−0.12	1.53			1.78	1.55		
	Mean monthly rainfall in first year	−2.52E−04	0.02	1.36E−04	.99	−0.03	0.02	2.04	.15
	*SD* monthly rainfall in first year	−0.001	0.01	0.01	.94	−4.70E−04	0.01	0.001	.97

**Figure 5:**
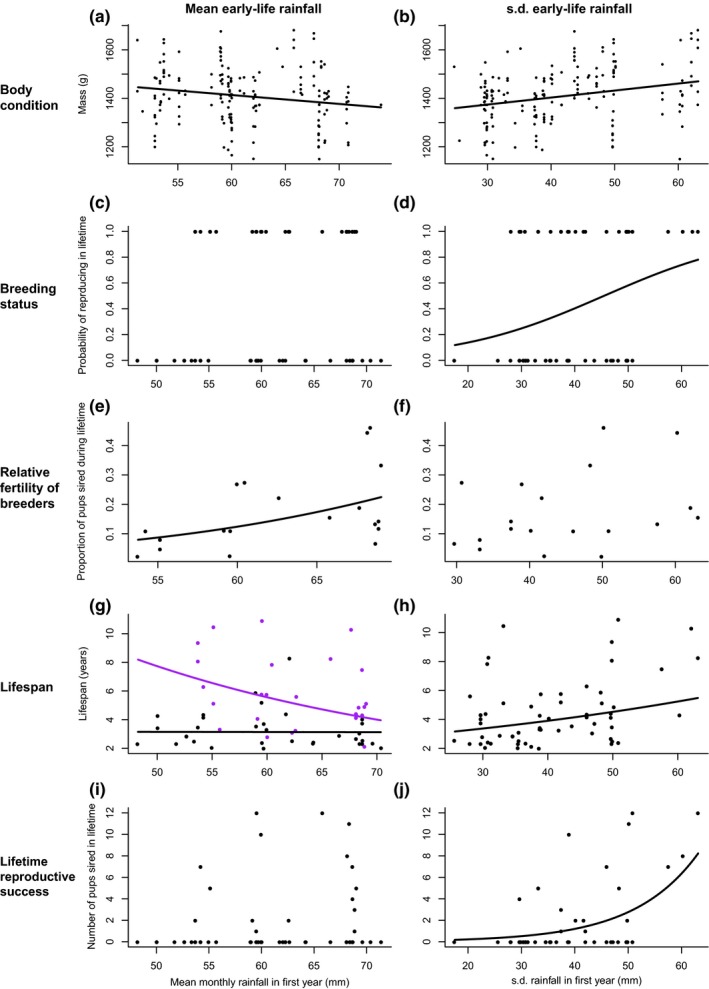
Male mongooses’ reproduction and survival and the mean (left‐hand panels) and variability (right‐hand panels) of rainfall in their first year. Panels show the effect on adult males’: mass (a, b), probability of siring at least one pup (c, d); in those that did sire a pup, the proportion of all pups born into their group that they sired (e, f); their lifespan (g, h); the total number of pups they sired in their lifetime (i, j). Lines show significant relationships predicted by models (see Table 3). Nonsignificant relationships are not plotted. In panel (g), the data and predicted relationships are split by into males who successfully reproduced in their lifetime (purple) and those who did not (black)

Early‐life conditions did have consistent effects on reproduction and survival in adult males (Table 3). Males born into years with more variable rainfall generally had better lifetime reproductive success and survival in later‐life (Figure 5, right‐hand panels), while the effect of the amount of first‐year rainfall indicated a potential life‐history trade‐off (Figure 5, left‐hand panels). Males that experienced more variable first‐year rainfall were heavier in adulthood (Figure 5b) and more likely to sire a pup during their lifetime (Figure 5d). The variability of first‐year rainfall did not affect successful males’ relative fertility (the proportion of all pups born into their group that they sired, Figure 5f), but males born in more variable rainfall years did live longer (Figure 5h). Ultimately, this translated into males born in more variable rainfall years having greater lifetime reproductive success (Figure 5j). Males born into years with less rainfall were heavier in adulthood (Figure 5a). However, in contrast to the effect of the variability of first‐year rainfall, the amount of first‐year rainfall did not affect the chance of a male siring at least one pup in their life (Figure 5c). However, if a male was a successful sire in their lifetime, those born into years with more rainfall had greater relative fertility (Figure 5e) but lived shorter lives (Figure 5g). This translated in the amount of first‐year rainfall having no effect on males’ overall lifetime reproductive success (Figure 5i).

**Table 3:** Models predicting the effect of early‐life ecological conditions on lifetime reproduction and survival. Parameter estimates (±standard errors) for models fitted to males and females are shown, along with likelihood‐ratio chi‐square statistics and *p*‐values. Significant effects of early‐life ecological conditions are highlighted in bold

Model predicting	Explanatory variable	Males				Females			
		β	*SE*	χ^2^	*p*	β	*SE*	χ^2^	*p*
Body condition	Intercept	1,451.14	113.43			1,211.50	303.06		
	Age	17.26	4.26	16.08	<.001	12.61	7.91	2.51	.11
	Mean monthly rainfall in first year	−**3.68**	**1.81**	**4.26**	**.04**	0.19	4.76	2.00E−05	.99
	*SD* monthly rainfall in first year	**2.89**	**1.05**	**7.49**	**.006**	0.81	1.32	0.43	.51
Successful reproduction in lifetime	Intercept	−7.10	4.63			2.96	4.72		
	Mean monthly rainfall in first year	0.06	0.05	1.76	.19	−0.04	0.07	0.40	.53
	*SD* monthly rainfall in first year	**0.07**	**0.03**	**5.41**	**.02**	0.01	0.04	0.06	.81
In successful reproducers, proportion of potential pups assigned in lifetime	Intercept	−6.77	1.59			5.02	3.30		
	Mean monthly rainfall in first year	**0.08**	**0.03**	**9.47**	**.002**	−0.08	0.05	3.44	.06
	*SD* monthly rainfall in first year	1.40E−03	0.01	0.01	.91	−**0.04**	**0.02**	**5.24**	**.02**
Lifetime reproductive success	Intercept	−3.61	3.58			0.46	4.28		
	Mean monthly rainfall in first year	0.01	0.06	0.02	.89	−0.01	0.06	2.49	.11
	*SD* monthly rainfall in first year	**0.08**	**0.04**	**5.14**	**.02**	−0.002	0.03	3.11	.08
Lifespan	Intercept	6.47	0.64			7.50	0.60		
	Mean monthly rainfall in first year	−3.12E−04	0.01			−0.01	0.01	0.37	.54
	*SD* monthly rainfall in first year	**0.015**	**0.005**	**7.27**	**.01**	−0.01	0.01	2.23	.14
	Successfully reproduced in lifetime (1/0)	2.53	0.95			0.54	0.13	18.03	<.001
	Successfully reproduced in lifetime ×								
	* *Mean monthly rainfall in first year	−**0.03**	**0.02**	**4.27**	**.04**	−0.02	0.02	1.02	.31
	* SD* monthly rainfall in first year	0.02	0.01	3.19	.07	−0.01	0.01	0.24	.62

The mean and variability of first‐year rainfall had almost no effects on female mass, reproduction and survival (Table 3). The one exception was that successfully breeding females born in years with more variable rainfall had a lower relative fertility, that is, were mother to a lower proportion of all pups born into their group during their lifetime (Table 3)

## 4 Discussion

**Figure 6:**
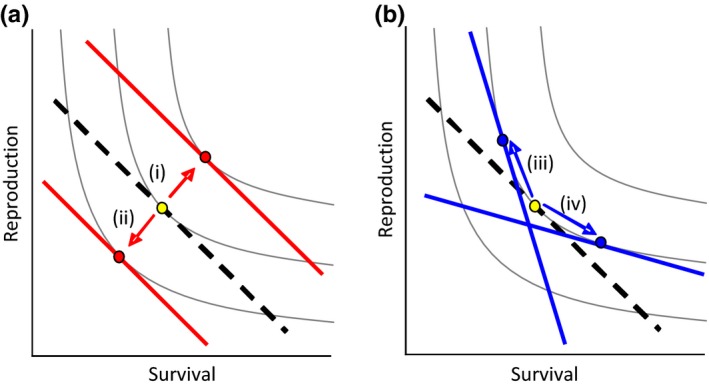
Graphical illustration of the hypothesized effect of changes in the (a) variability and (b) mean of early‐life rainfall on life‐history trade‐off and allocation patterns (after Saeki et al., 2014). The thin gray lines represent fitness isoclines along which all positions return an equal payoff. The thick dashed line represents the reference trade‐off slope for an individual (here straight lines for simplicity), and the yellow dot represents the reference optimal allocation of resources to survival and reproduction (where the trade‐off slope is tangential to the isocline). In panel (a), the thick red lines and dots show how an (i) increase or (ii) decrease in the variability of early‐life rainfall relaxes or increases the life‐history trade‐off constraints leading to higher or lower fitness payoffs. In panel (b), the thick blue lines and dots represent how an (iii) increase or (iv) decrease in mean early‐life rainfall leads to changes in an individual's trade‐off slope and optimal allocation of resources but no change in their overall fitness payoff

Early‐life ecological conditions had lifetime consequences for male banded mongooses. More variable early‐life conditions had positive effects on males’ lifespan and lifetime reproductive success, suggesting a relaxation of life‐history trade‐off constraints. In contrast, changes in mean early‐life conditions influenced males’ relative fertility and lifespan in opposite directions resulting in no overall effect on lifetime reproductive success, suggesting a life‐history trade‐off. In Figure 6, we use a simple graphical model (adapted from Saeki, Tuda, & Crowley, 2014) to illustrate these different effects of the variability and mean early‐life conditions. Unlike in males, females’ early‐life ecological conditions had almost no effect on patterns of survival and reproduction.

Males who experienced more variable rainfall in early‐life lived longer and had greater lifetime reproductive success. We have shown previously that more variable rainfall is associated with higher adult mortality in mongooses (Marshall et al., 2016), and our results here also indicate an association with lower food abundance suggesting that years with more variable rainfall are generally unfavorable. These findings, therefore, contrast with previous studies of wild animals showing that favorable early‐life ecological conditions have positive effects on survival and reproduction in later life (e.g., *Haematopus ostralegus* Van de Pol et al., 2006; *C. elaphus* Nussey et al., 2007; *F. auricularia* Wong & Kölliker, 2014). Evidence for later‐life fitness advantages of unfavorable early‐life ecological conditions has been shown in two wild bird systems, but this was based on a single measure of fitness (probability of breeding in *L. superbus* Rubenstein et al., 2016) or was found within an overall positive effect of early‐life ecological conditions on fertility and survival (*P. major* Wilkin & Sheldon, 2009). Increased adult survival was also shown in female roe deer (*Capreolus capreolus*) that survived periods of high juvenile mortality (Garratt et al., 2015). Our study uses multiple measures of fitness to directly link later‐life fitness advantages to unfavorable early‐life ecological conditions (rather than measures of early‐life mortality) in a wild mammal system. Moreover, our results show that these later‐life advantages are not explained by the impact of early‐life conditions on offspring survival (i.e., selection), parental investment, or the social environment as suggested previously (Garratt et al., 2015; Rubenstein et al., 2016; Wilkin & Sheldon, 2009).

Why might more variable ecological conditions be associated with increased fitness payoffs, without any apparent costs (Figure 6a)? Previous studies investigating the effect of the variability of early‐life ecological conditions on later‐life performance in a wild animal system are lacking. One possibility is that more varied early‐life conditions might influence individuals’ physiological or cognitive development such that they are able to cope with a greater range of environmental challenges in later‐life. This hypothesis is supported by the fact that, at our study site, years with greater variation in monthly rainfall values were also those with greater maximum and lower minimum monthly rainfalls. Furthermore, comparing the range of monthly rainfall values males experienced during early‐life to the range of monthly values they would experience during a typical male lifespan (42 months, Cant et al., 2016), those born in highly variable periods (the top third of early‐life *SD* rainfall values) experienced 93% ± 7% (mean ± *SD*,* n* = 50 males) of the typical lifetime range, while those born in low variability periods (the bottom third of early‐life *SD* values) experienced 63% ± 8% (*n* = 55 males). Our results do not support the possibility that a more variable early‐life rainfall conferred advantages through a more diverse diet. However, there are parallels between our hypothesis that early‐life variability may have beneficial impacts on physiological or cognitive development and findings from the medical literature. First, experiencing a wide variety of pathogens during childhood is recognized to be important in the development of the human immune system, with a lack of early‐life immunological challenges leading to inappropriate immune responses in adulthood and allergic diseases (the “hygiene hypothesis”: Strachan, 1989, 2000; Wills‐Karp et al., 2001; Yazdanbakhsh et al., 2002). Second, experiencing a variety of childhood psychological stressors is thought to have positive effects on some cognitive abilities in adults under stress (Frankenhuis & de Weerth, 2013; Frankenhuis et al., 2015). Finally, it may be that reduced early‐life food availability is beneficial. In laboratory models and humans, dietary restriction has repeatedly been shown to increase lifespan (e.g., Colman et al., 2009; Fontana et al., 2004; Masoro, 2006; McCay et al., 1935; Zhang et al., 2013). In support of this recent ecological studies have shown that restrictions on early‐life diet can have benefits such as reduced oxidative damage in zebra finches, *Taeniopygia guttata*, and wild yellow‐legged gulls, *Larus michahellis* (Noguera, Lores, Alonso‐Alvarez, & Velando, 2011; Noguera, Monaghan, & Metcalfe, 2015). The link between our hypothesis and these biomedical findings is speculative at present, but there is clearly much scope for research to elucidate the mechanisms underlying the early‐life effects reported here.

Mean early‐life ecological conditions, rather than having an effect on overall fitness payoffs, appeared to mediate a life‐history trade‐off (Figure 6b). Depending on mean conditions during development, males apparently adopted a “live‐fast, die‐young” or “live‐slow, die‐old” strategy and achieved similar lifetime fitness as a consequence. These findings are consistent with life‐history theory that individuals trade‐off allocating resources between somatic maintenance and reproduction (Kirkwood & Rose, 1991; Reznick & Yang, 1993; Zera & Harshman, 2001), and support the general hypothesis that conditions during development have an important influence on this life‐history resource allocation across an individuals’ lifetime (Gluckman et al., 2005; Monaghan, 2008; Nettle & Bateson, 2015; Taborsky, 2006). Nonetheless, empirical studies demonstrating the effect of early‐life conditions on life‐history trade‐offs are uncommon (Cartwright et al., 2014; Hammers et al., 2013), with many more studies showing directly positive (or negative) “silver‐spoon” effects of early‐life conditions on lifetime fitness (Hayward et al., 2013; Nussey et al., 2007; Rubenstein et al., 2016; Van de Pol et al., 2006; Wong & Kölliker, 2014). Our results provide evidence for both life‐history trade‐off and “silver‐spoon” type effects of early‐life ecological conditions. Poor average early‐life conditions shifted life histories toward the slower end of the survival versus reproduction trade‐off, while more variable early‐life conditions had beneficial effects on both survival and reproduction. This highlights the importance of considering the variability, as well as the average, of early‐life conditions in understanding their consequences for individuals’ lifetime fitness.

Why did early‐life ecological conditions only have consistent effects on males’, and not females’, later‐life fitness? Previous studies have suggested that sex‐differences in the effect of early‐life ecological conditions may be due to sex‐differences in: (1) body size (Ancona & Drummond, 2013; Kruuk et al., 1999; Millon et al., 2011), (2) the amount of care received during development (Kruuk et al., 1999; Rubenstein et al., 2016), (3) the effect of early‐life conditions on the development of reproductive organs (Wong & Kölliker, 2014) and (4) selection pressures for plasticity to later‐life conditions (Ancona & Drummond, 2013; Garratt et al., 2015; Wilkin & Sheldon, 2009). We are able to rule out explanations (1) and (2) here since banded mongooses show little sexual dimorphism and early‐life ecological conditions did not predict the amount of care pups received. We have no direct evidence to rule out explanation (3), but we consider it unlikely since female reproductive organs are more costly to develop and so this would predict later‐life fitness effects in females rather than males (as Wong & Kölliker, 2014 found), which is the opposite to what we found. There is, however, evidence to support explanation (4) that banded mongoose females are under greater selection to exhibit higher levels of plasticity to ecological conditions experienced in adulthood. Adult females’ mass and survival are more sensitive than males’ to ecological conditions experienced during adulthood (Marshall et al., 2016). Females in better condition are more fecund and have more competitive offspring (Hodge et al., 2009; Inzani et al., 2016), and they reduce their costly helping behavior more than males when food availability is lower (Bell, 2010). These effects are all likely to increase selection on females to adapt to the ecological conditions they experience during adulthood, masking any effects of the conditions they experienced in early‐life. In addition, females also start breeding at an earlier age than males (Cant et al., 2016) and are pregnant for an average of 30% of their adult lives (H. H. Marshall and M. A. Cant, unpublished data). While this last point may impair females’ ability to respond to changes in ecological conditions while they are pregnant (Marshall et al., 2016), it may increase the pressure to adapt to these changes outside of pregnancy. These results contribute to the growing picture that the effect of early‐life ecological conditions on later‐life fitness is often sex‐specific (Millon et al., 2011; Rubenstein et al., 2016) and highlights sex‐differences in plasticity to later‐life ecological conditions as a mechanism producing this sex‐specific effect (Ancona & Drummond, 2013; Wilkin & Sheldon, 2009).

Finally, despite the multiple effects of early‐life ecological conditions in later‐life, we found no immediate effects of early‐life conditions on social care received or offspring survival. There was a trend for males to be heavier at 1 year old after a more variable early‐life; however, this effect was a nonsignificant trend (*p* = .06) and so needs further investigation. This agrees with other studies showing that early‐life conditions can influence individuals in later‐life without having impacts on early‐life health or survival (Andrews et al., 2015; Rosa et al., 2014). For example, in the European starling, *Sturnis vulgaris*, competitive disadvantage during development does not affect chick growth or mass a year later, but disadvantaged birds experience greater telomere attrition as chicks (Nettle, Monaghan, et al., 2015) and, as adults, impaired flight performance and altered foraging and cognitive behavior (Andrews et al., 2015; Bateson, Emmerson, Ergün, Monaghan, & Nettle, 2015; Nettle, Andrews, et al., 2015; O'Hagan, Andrews, Bedford, Bateson, & Nettle, 2015). Such “cryptic” or “carry‐over” effects of early‐life conditions may be mediated by physiological mechanisms that only manifest themselves in later life (Andrews et al., 2015; Nettle, Monaghan, et al., 2015). A greater understanding of how environmental conditions during development affect individuals’ physiology may be key in understanding why these conditions influence individuals’ health, behavior and fitness in later‐life, with implications for evolutionary and biomedical science (Gluckman et al., 2005; Nettle & Bateson, 2015).

## Data Accessibility

The data used in this paper is available on Figshare at https://dx.doi.org/10.6084/m9.figshare.4591597.v1.

## Conflict of Interest

None declared.
